# Goblet cell interactions reorient bundled mucus strands for efficient airway clearance

**DOI:** 10.1093/pnasnexus/pgad388

**Published:** 2023-11-10

**Authors:** Meike F Bos, Anna Ermund, Gunnar C Hansson, Joost de Graaf

**Affiliations:** Institute for Theoretical Physics, Center for Extreme Matter and Emergent Phenomena, Utrecht University, Princetonplein 5, 3584 CC, Utrecht, The Netherlands; Department of Medical Biochemistry and Cell Biology, University of Gothenburg, PO Box 440, 405 30, Gothenburg, Sweden; Department of Medical Biochemistry and Cell Biology, University of Gothenburg, PO Box 440, 405 30, Gothenburg, Sweden; Institute for Theoretical Physics, Center for Extreme Matter and Emergent Phenomena, Utrecht University, Princetonplein 5, 3584 CC, Utrecht, The Netherlands

**Keywords:** lung clearance, bundled mucus strands, goblet cells, Langevin dynamics, bio-inspired model

## Abstract

The respiratory tract of larger animals is cleared by sweeping bundled strands along the airway surface. These bundled strands can be millimetric in length and consist of MUC5B mucin. They are produced by submucosal glands, and upon emerging from these glands, the long axis of the bundled strands is oriented along the cilia-mediated flow toward the oral cavity. However, after release, the bundled strands are found to have turned orthogonal to the flow, which maximizes their clearance potential. How this unexpected reorientation is accomplished is presently not well understood. Recent experiments suggest that the reorientation process involves bundled strands sticking to MUC5AC mucus threads, which are tethered to the goblet cells. Such goblet cells are present in small numbers throughout the airway epithelium. Here, we develop a minimal model for reorientation of bundled mucus strands through adhesive interactions with surface goblet cells. Our simulations reveal that goblet cell interactions can reorient the bundled strands within 10 mm of release—making reorientation on the length scale of the tracheal tube feasible—and can stabilize the orthogonal orientation. Our model also reproduces other experimental observations such as strong velocity fluctuations and significant slow-down of the bundled strand with respect to the cilia-mediated flow. We further provide insight into the strand turning mechanism by examining the effect of strand shape on the impulse exerted by a single goblet cell. We conclude that goblet cell–mediated reorientation is a viable route for bundled strand reorientation, which should be further validated in future experiment.

**Significance Statement**

Recent experiments have revealed that healthy mammalian lungs do not have a thick mucus layer covering the respiratory tract, as is the accepted picture informed by air–liquid interface cell studies. Instead, the larger airways possess submucosal glands that form mucus into bundled strands. Here, we therefore break with the tradition in microhydrodynamic modeling of mucociliary clearance, by instead focusing on the dynamics of bundled strands at the macroscale of the large airways. Our approach takes cues from traditional polymer-in-flow modeling and qualitatively recovers the main features of the experiments. This constitutes a meaningful step toward a full picture of how bundled strands mediate airway clearance, and it offers a new route toward analyzing the way in which diseases impact this mechanism.

## Introduction

Mucociliary transport (MCT) refers to a self-cleaning mechanism of the airways. The view has been that the airways are coated by an inhomogeneous viscoelastic mucus layer that is moved out of the lungs by the beating motion of cilia, which are grouped together on ciliated cells. Most of these experimental studies have been performed on air–liquid interface (ALI) cell cultures, where a single layer of epithelial cells is cultured on transwells. In this artificial system, there are no submucosal glands and large amounts of mucus are secreted by numerous goblet cells forming a mucus layer. This system has been used for studies of cilia beating, mucus transport, and mucus viscosity and to understand differences in MCT, when using cells derived from different diseases (1–4). ALI studies have largely directed the community's thinking on MCT.

**Fig. 1.**
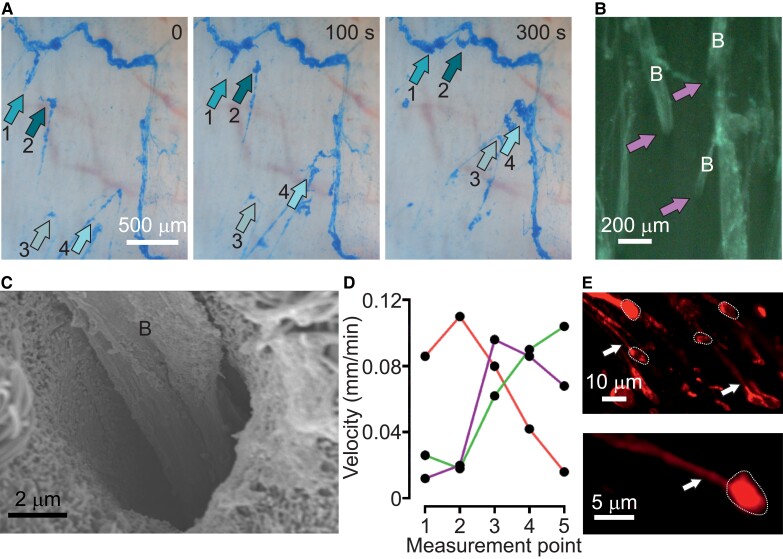
Experimental data. A) Snapshots from a 5-min movie (see Movie S1, time stamp at the top right) of live WT weaned pig airway stained with Alcian Blue. Numbered arrows follow the same bundled strand over the length of the movie (scale bar 500μm). Transport direction up in all images. B) Stereo-microscopic image of live WT weaned pig airway, stained with the lectin LTL labeled with fluorescein. Gland openings are indicated using arrows and bundled strands by the letter “B” (scale bar 200μm). C) Scanning electron micrograph of a gland opening with a single bundled strand (“B”; scale bar 2μm). D) Velocity of three bundled strands measured at five positions along the respective bundles illustrating that the bundled strands are retained at unpredictable points. E) Airyscan images of weaned pig airway surface goblet cells (dotted lines) secreting UEA1-stained mucus threads (mostly MUC5AC, white arrows). Scale bars 10 µm top, 5 µm bottom image.

However, experimental studies on healthy young pigs, with an airway architecture and submucosal glands similar to humans, reveal a different picture of normal MCT (5–7). It is now understood that normal, healthy lungs do not have a mucus layer covering the respiratory tract. Instead, larger airways have submucosal glands that form mucus (type MUC5B) into bundled strands that are several tens of micrometers in diameter (6, 8, 9) and potentially have lengths in the millimeter range. An example of bundled strands moving over the pig airway surface is shown in Fig. 1A (Movie S1). These observations come from experiments, wherein explanted pig distal trachea was mounted, the mucus was stained with the cationic dye Alcain Blue, and the sample was analyzed using video microscopy (6). Full experimental details are provided in the “Materials and Methods” section.

Recently, Fischer et al. (10) experimentally showed that these bundled strands are crucial in initiating the movement of larger inhaled particles. Bundled strands were argued to interact with each other and thereby form a complex network, which collects introduced debris (350 μm tantalum disks), and collectively provides sufficient pull to dislodge these. A key to the formation of a network is reorientation, as bundled strands emerge from submucosal glands with their long axis directed along the global flow (5, 6, 9–11; Fig. 1B and C]. In uniform Stokes flow, uniaxial objects are expected to remain parallel to the direction of motion and well separated. Nonetheless, further toward the larynx, bundled strands are observed with an orientation perpendicular to the flow (6, 10), as also shown in Fig. 1A (Movie S1). The processes by which this reorientation occurs remain poorly understood.

Experimental research has revealed three features of bundled strand motion that provide insight into the origin of reorientation. (i) Large bundled strands are observed to assume a zig-zag movement and appearance (9, 10, 12), see Fig. 1A (Movie S1). (ii) They are found to move slower than the background fluid that they are suspended in (6). (iii) The velocity by which bundled strands move is not constant (6), also see Fig. 1D. In previous observations by some of us (13), it was hypothesized that bundled strands reorient by temporarily attaching to MUC5AC mucus threads anchored in the goblet cells as shown in Fig. 1E. Observations (i–iii) align with this idea. Today, we do not understand the molecular details of how the bundled strands are pinned to the surface goblet cells. However, it is known that the threads secreted from surface goblet cells include the MUC5AC mucin and that these threads will coat the MUC5B bundles from the submucosal glands (6, 12). Imaging reveals mucins attached in the goblet cells with mucins extending out of the cells and coating the MUC5B bundled strands. Assuming that goblet cells interact with MUC5B bundled strands via MUC5AC threads, it is not obvious that this should reorient the bundled strands nor is it clear that this would stabilize perpendicular transport. Reorientation has not been directly observed, due to the experimental challenges in performing such a measurement.

Here, we therefore turn to numerical modeling. MCT has been investigated using simulations in the past (14, 15). This previous work has focused on the effect of ciliary beat frequency and global pattern, including metachronal waves, on mucus transport (16–23), as well as that of viscosity (17, 24–26) and viscoelasticity (27–33). However, neither mucus threads from goblet cells nor bundled mucus strands have been studied computationally, which thus requires a departure from established approaches.

### Numerical model

**Fig. 2.**
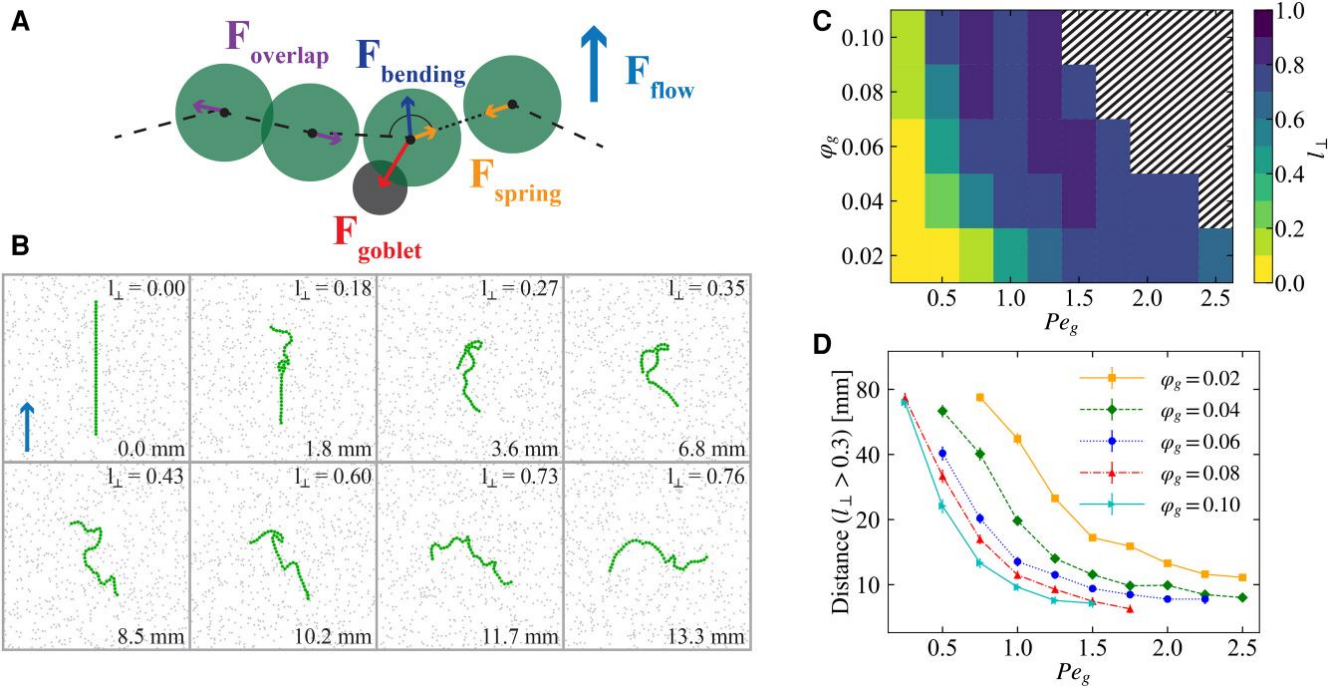
Numerical model and reorientation. A) Schematic of our numerical bead-spring model indicating the various forces present in the system. Beads (green disks) are connected by springs ($F_{\mathrm{spring}}$; orange arrows) and repel each other when they start to overlap ($F_{\mathrm{overlap}}$; purple arrows). The model bundled strand experiences bending forces ($F_{\mathrm{bending}}$; dark blue arrow) and is transported by a uniform fluid flow acting on each bead ($F_{\mathrm{flow}}$; light blue arrow). Last, the mucus threads tethered to goblet cells (gray disks) can form harmonic, breakable bonds with the beads ($F_{\mathrm{goblet}}$; red arrow). Full details are provided in the main text. B) Snapshots of a time series (Movie S2) showing a simulated bundled mucus strand (green) interacting with goblet cells (gray circles). The bundled strand starts with its long axis parallel to the flow (upward, blue arrow in the top-left panel). Time increases from left to right and top to bottom, here indicated by the distance (in mm) traversed by the strand's CoM (bottom-right corner). The relative amount of cross-section perpendicular to the direction of flow using $l_{⊥}$ is indicated in the top-right corner of each snapshot. The system is characterized by a ratio of sticking force to flow of $Pe_{g}=2.0$, a patch area fraction of $\varphi_{g}=0.04$, and a bending force expressed in terms of the flow strength of $Pe_{\mathrm{bend}}=0.3$. C) Goblet cell concentration ($\varphi_{g}$) and adhesion ($Pe_{g}$) state diagram of the fractional cross-section perpendicular to the flow ($l_{⊥}$), as indicated by the legend. We used a ratio of bending strength to flow of $Pe_{\mathrm{bend}}=0.3$. The striped part of the plot indicates phase points for which more than half of the simulated strands became pinned to the surface. D) Average distance traveled by strand's CoM to reach a perpendicular cross-section ratio of $l_{⊥}\geq 0.3$ as function of $Pe_{g}$, each point is averaged over 50 simulations, error bars (smaller than data points) indicate SEM.

We developed a minimal numerical model that captures the salient features of the observed bundled strand dynamics. In this model, motion is restricted to 2D, since we are interested in the large-scale movement of the bundled strands, which takes place along the lining of the airways. This lining consists of a thin fluid layer of 7 to 10μm in height (6, 34), which vertically confines the bundled strands, justifying our approximation. We drew inspiration from the bead–spring polymer literature (35–37) in representing the MUC5B mucus bundles as soft disks connected by springs as shown schematically in Fig. 2A. The bundled strands have been estimated to be made up of 1,000 to 5,000 linear parallel MUC5B polymers (6, 7). These have not been studied biophysically, in contrast to other thinner mucus structures (12). Given that the biophysical properties of the bundled MUC5B strands are presently unknown, we chose to use an established, generic bead–spring model, see below and the “Materials and Methods” section. The bundled strand's attachment to the surface cells has been proposed to be mediated via MUC5AC mucin, but the numbers of molecules involved at each attachment site and the forces involved are not known. We therefore make a minimal modeling assumption, see below, on the nature of these interactions. The beads of our bead–spring bundled strand are disks with diameter of $\sigma_{b}=25\;\mu m$, meaning that our typical value of 41 disks corresponds to a bundled strand that is 1.025mm in length. Goblet cells have a diameter of approximately 10 μm (38, 39), which we represent by disks with diameter of $0.5\sigma_{b}$ in our model (the exact diameter will not prove important). These model goblet cells are placed randomly on the surface in a nonoverlapping way using a separate simulation; see the “Materials and Methods” section. We used a range of goblet cell area fractions $\varphi_{g}\in [0.02,0.1]$; the exact value in biological systems is not known, but area fractions of around 5% were measured in trachea and bronchi of rats by Mercer et al (38).

An overdamped Langevin equation describes the bead dynamics, for which three *internal* forces set the properties of our bundled strand: spring forces between the beads set the elasticity, bending forces set the persistence length, and repulsive forces that mimic excluded volume interactions between different parts of the model bundled strand. As stated above, there is no biophysical information available on the rheological properties of the bundled strands. The current modeling is therefore based on standard choices from the polymer literature; this aspect may be refined when further experimental data on the bundled strands become available. The model strand interacts with its environment via two *external* forces: a constant drag force that captures the effect of the overall flow toward the oral cavity on the bundled strand, and breakable harmonic bonds that represent adhesive interactions between the bundled mucus strands and mucus threads tethered to goblet cells. To the best of our knowledge, there are no quantitative data on the physical properties of MUC5B bundled mucus strands, which we could use to directly determine the parameters in our model. Instead, we estimated values based on qualitative observations, which are informed by exploratory simulations that probe the parameter ranges for which our model is stable. Full details are provided in the “Materials and Methods” section, and we will cover the strengths and limitations of our modeling in the “Discussion” section.

The behavior of our model bundled mucus strands is dictated by the relative strength of the involved forces. We introduce modified Péclet numbers to characterize these ratios. They express the strength of a force (labeled *X*) of interest ($F_{X,i}$), acting on the *i*th bead, in terms of the drag force on a single bead ($F_{\mathrm{flow},i}$), due to the background flow: $Pe_{X}=F_{X,i}/F_{\mathrm{flow},i}$. For the bending stiffness, we used a value of $Pe_{\mathrm{bend}}=0.3$ throughout, which provides reasonable shape fluctuations of the strand; we motivate our choice further in the “Materials and Methods” section. We consider goblet cell interaction strengths in the range of $Pe_{g}\in [0.25,2.5]$. The strength of this interaction is not known experimentally, and therefore it must be a free parameter in our modeling.

## Results

### Reorientation of modeled bundled strands

Our bundled strands start parallel to the direction of the flow. This represents the experimentally observed configuration upon release from a gland (5). In many cases, our model bundled strands subsequently turn perpendicular to the flow. Figure 2B shows a representative time series of a reorientation event (Movie S2) for $\varphi_{g}=0.04$ and $Pe_{g}=2.0$. First, the front of the bundled strand buckles and folds, after which it elongates in the direction perpendicular to the flow. Finally, a steady state is reached where the bundled strand is oriented perpendicular to the flow, and there are only minor fluctuations in its shape. Our numerical analysis thus shows that breakable adhesive interactions can reorient a model bundled strand starting from a parallel orientation to a stable perpendicular orientation with respect to the flow.

For our mechanism to be biologically relevant, the length scale on which the turning event takes place is crucial. That is, the bundled strand must reorient fast enough to effectively contribute to clearance before it has reached the larynx. We measured $l_{⊥}$ to quantify this reorientation; this represents the perpendicular cross-section normalized by the length of the strand. We report on the length of travel, rather than a time scale here, because the former is insensitive to our choices of hydrodynamic coupling between the model bundled strand and the uniform background flow. The center-of-mass (CoM) distance traveled by a strand (starting with $l_{⊥}=0$) before reaching $l_{⊥}>0.3$ proved a reasonable measure of orthogonalization. We established this number by inspecting a number of configurations; our result does not qualitatively depend on the exact number. For the reorientation process in Fig. 2B, we find a value of approximately 6mm of CoM travel (lower right corner) from values of $l_{⊥}$ (upper right corner). From experiments, we know that strands exit the gland duct in which they are formed, oriented parallel to the flow toward the oral cavity, and do not reorient in the process of becoming disconnected from the duct. They are later observed to move over the surface in an orthogonal configuration, in the region where a mucus network starts forming. This means that reorientation does not happen instantaneously, yet it must occur on a length scale that is much smaller than the total length of the trachea. We emphasize that the distance of 6 mm satisfies this criterion. The turning length may be shifted by modifying the strand and surface parameters, as we explore in the next section.

We verified the robustness of our result by analyzing bundled strand dynamics as function of $\varphi_{g}$ and $Pe_{g}$, taking an average over 50 simulations per state point. Figure 2C shows the average $l_{⊥}$ after $2,000\sigma_{b}/v_{\mathrm{flow}}$. For our modeling choices, this time corresponds to at most 50mm of travel. This distance was found to be a reasonable time scale to obtain steady state configurations in a large area of the state space; Fig. S1 shows the behavior for additional times. However, for sufficiently high $\varphi_{g}$ and $Pe_{g}$, the strands become trapped, as indicated by the hashing in Fig. 2C. Figure 2D shows the averaged distance before orthogonalizations as a function of $Pe_{g}$ for several $\varphi_{g}$. Increasing $Pe_{g}$ always strongly reduces the travel distance; above $Pe_{g}=1.5$ reorientation can happen within 10mm. Similarly, the distance traveled decreases by increasing $\varphi_{g}$. Note that some curves in Fig. 2D are truncated as the strands become trapped.

### Shape and velocity fluctuations

**Fig. 3.**
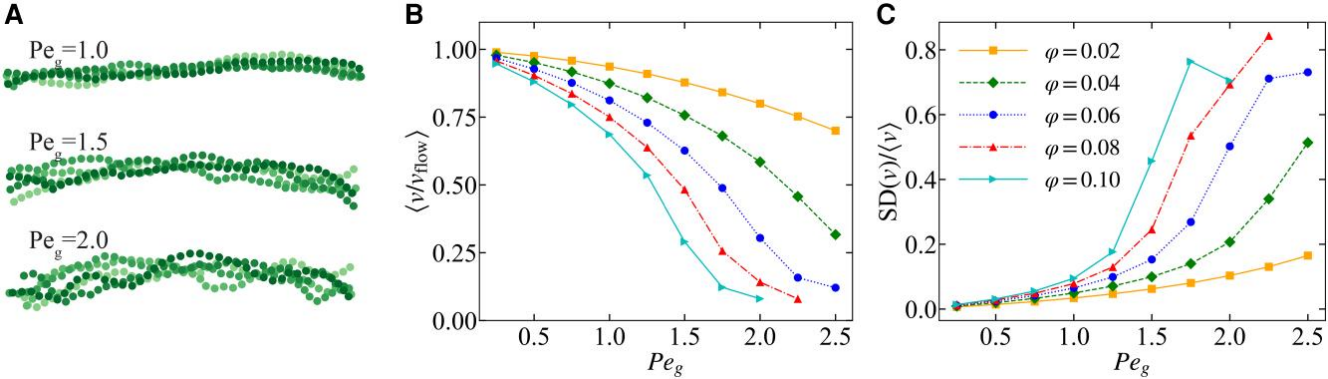
Properties of the steady-state perpendicular motion. A) Superposition of five simulation snapshots (taken 100Δt apart) for three values of the goblet cell interaction strength ($Pe_{g}$); time increases from lighter to darker shadings of green. We used goblet cell area fraction $\varphi_{g}=0.04$ and bending potential $Pe_{\mathrm{bend}}=0.3.$ B) The mean velocity ⟨v⟩ averaged over 50 simulations of a perpendicular orientated strand (expressed in the flow speed $v_{\mathrm{flow}}$) in simulations as function of $Pe_{g}$ for range of $\varphi_{g}$. The error bars (smaller than the data points here) represent the SEM. C) SD of the velocity expressed in terms of ⟨v⟩ for same data as in B). The error bars represent the SEM.

Breakable adhesive surface interactions are hypothesized to not only play a role in the reorientation of bundled strands but also in setting their zig-zag shape and variable velocity. We vary $\varphi_{g}$ and $Pe_{g}$ to investigate these features in our model; Fig. 3A shows three snapshots for a system with $\varphi_{g}=0.04$ and three different values of $Pe_{g}$. With increasing $Pe_{g}$, the zig-zag shape becomes more pronounced and qualitatively mimics the one observed in experiments as shown in Fig. 1B. In Fig. S2, we quantified the fluctuations in the shape using the normalized parallel cross-section ($l_{∥}$), defined in analogy to $l_{⊥}$, as function of system parameters. Figure 3B and C shows the mean velocity ⟨v⟩ and the relative fluctuations in velocity (measured using the SD), respectively, as function of $Pe_{g}$ for different values of $\varphi_{g}$.

We find that ⟨v⟩ decreases with increasing $Pe_{g}$ and $\varphi_{g}$, while the relative fluctuations increase (though the fluctuations are nearly independent of $\varphi_{g}$ above $\varphi_{g}>0.02$). In all cases, the bundled strand moves slower than the background flow. The experiments report a mean speed of $v\approx 0.3v_{\mathrm{flow}}$ (6), which corresponds to $Pe_{g}>1.5$ and $\varphi_{g}>0.02$ in our model. Examining the relative fluctuations, we find fluctuations above 10% of the mean value for $Pe_{g}>1.5$, increasing up to fluctuations of 80% of the mean value. This is commensurate with the large velocity fluctuations observed in experiments, as shown in Fig. 1D, though the data are not of sufficient quality to make a quantitative comparison.

### Understanding bundled strand reorientation

**Fig. 4.**
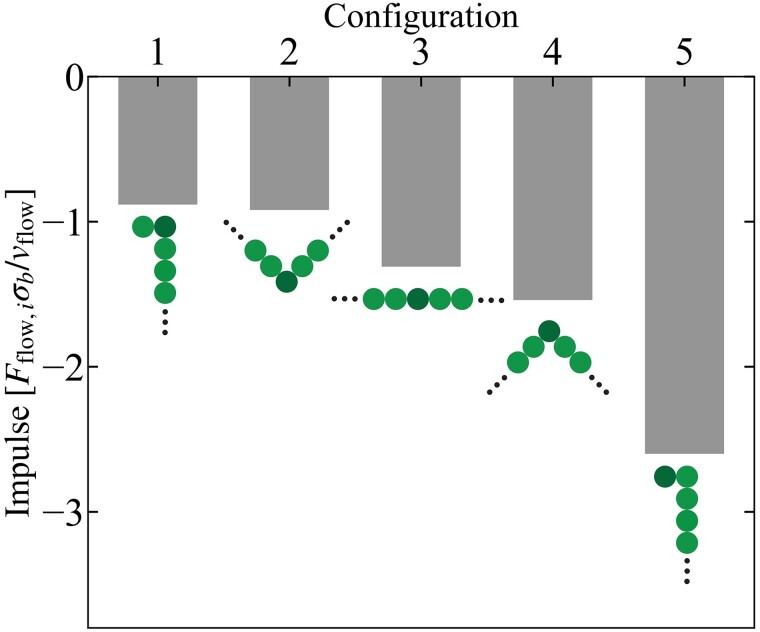
Impulse on bead interacting with goblet cell as function of the configuration of the strand. We show five idealized configurations, labeled by the numbers 1–5 (to which we refer in the main text), ordered from left to right with increasing strength of impulse. The schematics show 5 beads of a bundled strand comprising 41 beads, the black dots indicate how the strand continues. The dark-colored bead indicates the bead interacting with a goblet cell. The fluid flow points in the upward direction. The impulses are reported for a system with $Pe_{g}=1.5$ and $Pe_{\mathrm{bend}}=0.3.$

Next, we provide physical intuition for why the perpendicular part of the strand grows over time and the perpendicular orientation is stable. To gain insight in the reorientation mechanism, we isolated the effect of a single goblet cell interacting with several idealized configurations. These idealizations represent (parts of) the bundled strand configurations found during and after reorientation and allow us to study the effect of goblet cell interaction on (part of) a model bundled strand in a controlled manner. We measured the modeled forces experienced by a specific bead interacting with the single goblet cell and converted this into a goblet cell impulse; the force integrated over the total interaction time. The configurations and corresponding impulses are shown in Fig. 4. All impulses are negative, as the goblet cells hold the bundled strand back with respect to the fluid flow.

We find a strong dependence of the felt impulse on the local shape of the strand. The way in which the impulse depends on the local shape provides insight in both the reorientation and the stability of the perpendicular orientation. Comparing the magnitude of the impulse, the reorientation of the strand can be understood as follows. The front of a parallel, detached bundled strand is first to make contact with a goblet cell. When it connects, the tip buckles, as represented by configs 1 and 5. The now partially buckled strand interacts with subsequent goblet cells. It can either bend back (config 1) or buckle further (config 5). The impulse that comes from interacting with the off-parallel part of the strand is largest, when comparing configs 1 and 5. This suggests that growth in the orthogonal direction will probabilistically win from events that would bend the tip back parallel to the flow. This process repeats itself, until a perpendicular orientation is achieved.

The stability of the perpendicular orientation can be understood by comparing the impulses corresponding to configs 2 to 4. Once a part starts trailing behind (config 2), a goblet cell will pull it back less than a part that is ahead of the center (config 4). This causes leading parts of a bundled strand to exchange with trailing parts, which stabilizes the perpendicular orientation. The bead chain nature of the bundled mucus strand in our model will not affect the shape dependency of the impulse. The fact that the forces work on the center of the beads can be thought of as MUC5AC threads sticking to specific points on the bundled strand.

## Discussion

Our work is a first step toward an explicit model to understand the effect of mucus bundled strand movement in MCT. Here, we have proposed a minimal model that considers the dynamics of MUC5B bundled strands interacting with goblet cells via MUC5AC threads, which can be numerically solved. From a physics perspective, it is not obvious that such interactions would facilitate reorientation and stabilization of a perpendicular orientation, as mentioned in the “Introduction” section. In the following, we will provide further insight into our modeling choices, such that the limitations of our study may be appreciated.

At present, we have used a free-draining approximation for the cilium-induced flow. In reality, the bundled strands are partially immersed in a thin layer of a complex fluid, which is moved from below by the collective beating action of ciliated cells. There are local minor variations in beating direction, resulting in small-scale heterogeneities of the fluid flow (40–42). In addition, there is a large-scale drift toward the ventral side of the trachea (8). In our modeling, we have neglected these heterogeneities, as well as hydrodynamic coupling between the beads. In general, the hydrodynamic coupling between an object and the fluid in which it is embedded influences the orientation of the object. In strongly confined fluid configurations, like a Hele–Shaw geometry—reminiscent of the confinement experienced by the bundled strands—this coupling can cause elongated *rigid* objects with one symmetry axis to turn their long axis parallel to the direction of flow (43–46). Depending on the properties of the flow, flexible filaments show a wide range of orientational dynamics (37). The hydrodynamic effect on the orientation of the mucus bundles in our idealized geometry and that of the experiment is not known. The multiscale nature of the bundled strand clearance problem (≈1mm bundled strand moving over ≳10mm of airway, covered by 10μm ciliated cells that generate local flows) makes it computationally challenging to study the impact of fluid dynamics on a flexible strand. A full analysis of the impact of local flows is therefore left to future work. Nonetheless, we are confident that goblet cell interaction is dominant, as we require $Pe_{g}≳1.5$ to account for slow-down and the zig-zag configuration. This supports our assessment that sticking to goblet cells reorients the bundled strands in the experiment.

Taking a broader perspective on the behavior of our model bundled strands, we make an analogy to the pinning transition from the field of elastic manifolds driven through random media (47, 48). Pinning occurs when the relative strength of the random interactions balances the driving force on the elastic interface. When approaching the pinning transition from above, the velocity of the interface slows down, until it comes to a complete standstill at the transition. Our model exhibits these features, as can be seen in Fig. S3: bundled strands become trapped (or by analogy “pinned”) as $Pe_{g}$ increases, with the velocity significantly decreasing upon approaching this state. Full characterization of the pinning transition focuses on its critical exponents, which establish the universality class. Our bundled strands are typically too short to perform the required analysis, and we therefore cannot complete the analogy. However, based on our numerical results, we consider it likely that bundled strand MCT operates close to the “pinning transition.” In our simulations of individual bundled strands, pinning would lead to failed clearance. However, in the experimental setting, multiple strands could collide, by which trapped ones may be pulled loose.

In the biological system, interaction between two mucins is likely mediated by von Willebrand D or CysD domain interactions (13). However, how the secreted mucin can be attached in the goblet cell is not understood today. Studies of mucus in the intestine show that the mucus in the normal colon is attached but not attached in the small intestine. Interestingly, in the small intestine, the activity of a protease is involved in regulating this detachment in a well-controlled way (49). The von Willebrand factor (VWF), a protein involved in blood coagulation, is structurally closely related to the mucins (50). The VWF is secreted from endothelial cells into long linear polymers, just as the MUC5B mucin. The VWF has a hidden cleavage site for the ADAMTS13 protease (51, 52). This cleavage site is only exposed after sufficient mechanical pulling of the VWF to unfold the protein and allowing ATAMTS13 to reach its cleavage site and disrupt the VWF. Whether protease activity is required in the airways or if release from noncovalent interaction is the key factor remains to be explored. Nonetheless, the current in-silico modeling shows that transient pinning of the bundled strands to the surface epithelium is a sufficient and likely explanation for how MUC5B bundled strands reorient.

Our model captures the main feature that the bundled strands move over the tracheal surface in live tissue. This is important, as current understanding could not explain how a linear structure can be transported perpendicular to rather than parallel with the liquid flow. The transient interactions are likely explained by MUC5AC forming links between the bundled strands and surface goblet cells, as observed experimentally (6, 7). The number of anchoring molecules and the attach/detachment forces involved are presently unknown, making more refined simulations impossible. Once more molecular and biophysical information are available, the limitations of our modeling can be overcome, and a more refined picture of the dynamics of and clearance by bundled strands can be established. Such investigations can build upon the foundation we have provided in this work.

## Materials and methods

### Animals

Experimental protocols were in accordance with the EU Directive 2010/63/EU for the care and use of laboratory animals, the NIH, and ARRIVE guidelines. Ethical permission (2937/2020) was approved by the Swedish Laboratory Animal Ethical Committee in Gothenburg, Sweden. Female weaned pigs (*Sus scrofa domesticus*) weighing 8–12 kg were acquired from a local farm, housed according to Swedish legislation and allowed to acclimatize for 5 days. Sedation was performed with an intramuscular injection of 0.6 mg/kg Dexdomitor (Orion Pharma, Danderyd, Sweden) and 0.03 mg/kg Zoletil (Virbac, Kolding, Denmark). The pigs were killed by intravenous installation of 200 mg/kg Allfatal (Omnidea, Stockholm, Sweden). Death was ensured by lack of heart sounds and circulatory arrest.

### Live explant video microscopy

The live distal trachea and proximal primary bronchi were opened along the dorsal smooth mounted with the mucosal surface exposed in a Petri dish coated with Sylgard 184 Silicone Elastomer (Dow Corning, Wiesbaden, Germany) using insect pins (Cat# 26000-25, Agnthos, Lidingö, Sweden). The bundled mucus strands were stained with 0.4 mM Alcian Blue 8GX pH 7.4 (Cat# A5268, Sigma-Aldrich, St. Louis, MO) or 5 µg/ml fluorescein-labeled *Lotus tetragonolobus* (LTL) lectin (Cat# FL-1321-2, Vector Laboratories, Burlingame, CA) dissolved in 500 µl of oxygenated (95% O_2_, 5% CO_2_) Krebs glucose buffer (116 mM NaCl, 1.3 mM CaCl_2_, 3.6 mM KCl, 1.4 mM KH_2_PO_4_, 23 mM NaHCO_3_, 1.2 mM MgSO_4_, 10 mM d-glucose, 5.7 mM pyruvate, 5.1 mM glutamate, pH 7.4) and gradually heated to 37°C. The dish was placed on a table with a 20° incline to assure ALI and mucus transport against gravity. Time-lapse recordings and images were acquired through an SMZ18 stereo microscope (Nikon, Tokyo, Japan) and white light (Photonics, Pittsfield, MA) or a CoolLED pE-300ultra light source (CoolLED, Andover, UK) using a 5.9-megapixel color CCD camera (DS-Fi3, Nikon, Tokyo, Japan) and NIS elements software (RRID:SCR_014329, Nikon, Tokyo, Japan).

### High-resolution imaging

Mid tracheas from weaned pigs were dissected into 1 cm pieces, opened, and pinned mucosal side up with insect pins (Cat# 26000-25, Agnthos) to Petri dishes coated with Sylgard elastomer (Dow Corning). To visualize the mucus threads, DyLight 649–labeled *Ulex europaeus* agglutinin (UEA1) (Cat# DL-1068-1, Vector Laboratories) was dissolved at 5 µg/ml in Krebs glucose buffer, pH 7.4 (buffer composition as above) and added to the tissue. Live explants were incubated for 10 min at ambient temperature with 50 µl lectin solution, and 2 ml Krebs glucose was added before imaging. Images were acquired using a Zeiss LSM900 Axio Examiner Z1 microscope with the Airyscan two-imaging system and Zen blue software (RRID:SCR_013672, Carl Zeiss, Oberkochen, Germany). After acquisition, Airyscan images were processed using standard Airyscan processing algorithms.

### Electron microscopy

Pieces of the trachea two to three cartilage rings in length from weaned pigs were fixed in modified Karnovsky's fixative (2% paraformaldehyde, 2.5% glutaraldehyde in 0.05 M sodium cacodylate buffer, pH 7.2) for 24 h at 4°C. Postfixation was performed in 1% OsO_4_ at 4°C three times with intervening 1% thiocarbohydrazide steps. The samples were dehydrated with increasing concentrations of ethanol followed by hexamethyldisilazane that was allowed to evaporate. Samples were mounted on aluminum specimen pin stubs (Cat# AGG301, Agar Scientific, Stansted, Essex, UK) with carbon tabs (Cat# AGG3347N, Agar Scientific) and conductive silver paint (Cat# 16040-30, Ted Pella, Redding, CA). To decrease charging, samples were sputter coated with palladium before imaging at 3 kV in a field emission scanning electron microscope (Zeiss Leo Ultra 55, Carl Zeiss).

### Bundled mucus strand model

We modeled the bundled mucus strand as beads connected by springs. The beads have a diameter of $\sigma_{b}$, which we use as the unit of length in our simulations. The strand is made of a single chain of beads, and thus its diameter is also given by $\sigma_{b}$. The main text provides relative values and justification. The dynamics of the beads in our model is captured by an overdamped Langevin equation, where the velocity $v_{i}$ of bead *i* reads:


(1) $$\xi v_{i}=F_{\mathrm{int},i}(r_{i})+F_{\mathrm{ex},i}(r_{i}).$$

Here, the sum of the internal and external forces on bead *i* are given by $F_{\mathrm{int},i}\;$ and $F_{\mathrm{ex},i}$, respectively. We chose $\xi =3\pi \eta \sigma_{b}$ for the Stokes friction coefficient of the bead, with *η* is the viscosity of the fluid. That is, here, we approximate *ξ* by its bulk value for a Newtonian fluid acting on a perfect sphere. The true value of the friction in experiment is set by confinement effects, porosity of the strand, and the non-Newtonian character of the suspending fluid. It is crucial to understand that in our minimal model, the beads should experience some form of overdamped motion. However, the exact nature of the friction coefficient may be absorbed in a rescaling of another quantity, e.g. the time. This is one of the reasons why we report distance traveled by model bundled mucus strands in our analysis of their dynamics, rather than time traveled. The former quantity is not sensitive to the specific choice of friction, within our model.

The behavior of the strand is captured by three types of internal forces: (i) finitely extensible nonlinear elastic (FENE) springs connect the beads, giving rise to the elasticity of the bundled mucus strand; (ii) a bending force imposes the elongated shape of the strands, as observed experimentally; and (iii) a separation-shifted Lennard Jones (SSLJ) force between beads models excluded volume interactions. We discuss all contributions in detail below.

The corresponding potential for the FENE springs, connecting the beads, is given by [see Sadler (52)].


(2) $$U_{\mathrm{spring}}=\overset{N_{b}-1}{\underset{i=1}{\sum }}-\frac{1}{2}k_{\mathrm{spring}}\Delta r_{\max}^{2}\log\;\left[1-{\left(\frac{r_{i,i+1}-r_{0}}{\Delta r_{\max}}\right)}^{2}\right],$$

where $r_{ij}=|r_{i}-r_{j}|$ is the distance between the two beads; we only coupled successive beads *i* and i+1 here. The equilibrium length of the spring is given by $r_{0}$, and the maximum deviation from this equilibrium is given by $\Delta r_{\max}.$ That is, the maximum/minimum separation between successive beads is given by $r_{0}\pm \Delta r_{\max}$. In our simulation, we used $r_{0}=\sigma_{b}$ and $\Delta r_{\max}=0.8\sigma_{b}$. The FENE springs limit the extension of the total strand, which is desired to (i) stabilize the simulation and (ii) account for the limited extension of real MUC5B bundled strands.

In the experimental system, the bundled mucus strands keep their elongated shape while moving over the surface, indicating that there is some nonzero persistence length. We modeled this by introducing a bending potential, which favors a straight configuration of the strand (53). For the bending force on bead *i*, we use the potential of the form:


(3) $$\begin{array}{rl} U_{\mathrm{bending}} & =\overset{N_{b}-1}{\underset{i=2}{\sum }}\;k_{\mathrm{bend}}(1-\cos\;\theta_{i})\\ & =\overset{N_{b}-1}{\underset{i=2}{\sum }}\;k_{\mathrm{bend}}\left(1-k_{\mathrm{bend}}\frac{r_{i,i-1}\cdot r_{i,i+1}}{\left|r_{i,i-1}||r_{i,i+1}\right|}\right), \end{array}$$

where in the second equality, we introduced the shorthand notation $r_{i,i\pm 1}=r_{i}-r_{i\pm 1}$. The stiffness of the strand is set by the bending constant $k_{\mathrm{bend}}$. The angle $\theta_{i}$ is the angle of the vector $r_{i+1}-r_{i}$ and $r_{i}-r_{i-1}$.This angle is 0π, when the strand is straight locally. Experimentally, only the persistence length on a single mucin-level is known (54), and the persistence length a of bundled MUC5B mucus strand has not (yet) been measured. The value of $Pe_{\mathrm{bend}}=0.3$ is based on exploratory simulations, wherein we varied the bending stiffness. In these simulations, we found that a nonzero bending stiffness allows the model bundled strand to maintain a more elongated shape. For bending stiffnesses greater than $Pe_{\mathrm{bend}}=0.3$, our model bundled strands’ zig-zag shape becomes rounded and no longer mimics the one observed experimentally. We want to emphasize that for all values of bending stiffnesses that we used, we found reorientation on a length scale much smaller than the total length of the trachea. In Fig. S4, we show the results of these exploratory simulations for completeness.

The bundled mucus strands take up a finite volume, for which we introduced excluded volume interactions between the beads. We used a SSLJ pair potential (53), which captures the quasi-2D nature of the system. Although we simulate our system in 2D, in reality—if the forces become large enough—(parts of) bundled mucus strands can roll over each other in the direction perpendicular to the surface. The SSLJ potential allows for this because the interaction potential is finite at zero separation, and we used the following form of the SSLJ potential (55):


(4) $$U_{\mathrm{overlap}}=\overset{N_{b}}{\underset{i=1}{\sum }}\;\overset{N_{b}}{\underset{j>i}{\sum }}\;\{\begin{array}{c} 4E_{r}(\alpha^{12}{\left(\alpha^{2}+r_{ij}^{2}\right)}^{-6}-\alpha^{6}{\left(\alpha^{2}+r_{ij}^{2}\right)}^{-3}),\;\mathrm{if}\;r_{ij}<\sigma_{b},\\ 0,\;\mathrm{if}\;r_{ij}\geq \sigma_{b}, \end{array}$$

where $r_{ij}$ is the distance between two beads *i* and *j*. The scaled diameter is given by $\alpha =\sigma_{b}/\sqrt{2^{1/3}-1}$. The potential has a maximum repulsive value at r=0 of $U_{\mathrm{SSLJ}}(0)=E_{r}$.

In our model, we consider two external forces working on the bundled mucus strand. First, the strand is embedded in a fluid layer. This fluid layer moves with a mean velocity $v_{\mathrm{flow}}$ toward the oral cavity, due to the beating motion of cilia on ciliated cells. The strand is dragged along by this flow. Second, the strand can stick to mucus threads tethered to goblet cells at the surface. We explain in detail how we modeled both external forces below.

The force due to the background flow is modeled as a drag force on each bead,


(5) $$F_{\mathrm{flow},i}(r_{i})=\xi v_{\mathrm{flow}},$$

where we used $\xi =3\pi \eta \sigma_{b},$ the Stokes friction coefficient of the bead in a bulk fluid. We approximate the fluid flow as uniform and having a constant velocity: $v_{\mathrm{flow}}=v_{\mathrm{flow}}\hat{y}\;$ where $v_{\mathrm{flow}}$ is the average speed of the flow and the direction of the oral cavity $\left(\hat{y}\right)$. We have commented on the limitations of this approximation above.

We modeled the trapping of the bundled MUC5B strands to MUC5AC threads tethered to goblet cells by introducing a bond between the center of a goblet cell on the surface and the center of a bead moving over the goblet cell. The fact that the forces work on the center of the beads can be thought of as MUC5AC threads sticking to specific points on a bundled strand. This bond is only formed if the distance between the center of the bead and the center of the goblet cell is less than a minimal distance $r_{\mathrm{bond}}$; if the bond extended above $r_{\mathrm{bond}}$, it pulls on the strand with a Hookean spring force. The bond is stretched until a maximum distance $r_{\max}$, or equivalently maximum force $F_{g,\max}$ is reached, after which the bond breaks. By introducing an asymmetry between the forming and breaking of bonds, we mimic the fact that the mucus threads can adhere to the strand, and when the strand moves along these bonds extend until they break. Note that other mechanisms of breaking in the experimental system are also possible; the nature of the bonding remains to be settled. By making the force zero when the distance between the bead and goblet cell is smaller than $r_{\mathrm{bond}}$, we simulate that the mucus threads do not exert a force if they are not extended. We can summarize the goblet interaction potential in the following expression:


(6) $$U_{\mathrm{goblet}}=\overset{N_{b}}{\underset{i=1}{\sum }}\;\overset{N_{g}}{\underset{n=1}{\sum }}\;\{\begin{array}{c} 0,\;\mathrm{if}\;|r_{i}-r_{g,n}|<r_{\mathrm{bond}},\\ \frac{1}{2}k_{g}{\left(|r_{i}-r_{g,n}|-r_{\mathrm{bond}}\right)}^{2}\;\mathrm{if}\;\mathrm{bon}d_{i,n}=1\;\mathrm{and}\;r_{\mathrm{bond}}\;<|r_{i}-r_{g,n}|\;<r_{\mathrm{break}},\\ 0\;\mathrm{otherwise}. \end{array}$$

where $N_{g}$ denotes the number of goblet cells in simulations. $\;\mathrm{bon}d_{i,n}$ is 0 if there is no bond between bead *i* and goblet cell *n* and 1 if there is a bond between *i* and *n*. The strength of the interaction is set by the spring constant $k_{g}$. In our simulations, we use $r_{\mathrm{bond}}=0.5(\sigma_{g}+\sigma_{b})$ and $r_{\mathrm{break}}=2r_{\mathrm{bond}}=(\sigma_{g}+\sigma_{b})$, where $\sigma_{g}$ is the diameter of the goblet cell. Here, we use $\sigma_{g}=0.5\sigma_{b}$. For details on the simulation setup and mathematical definitions of measured quantities, see Supplementary material online. In Table S1 the length scales for all interaction forces in our model are given, as well as the range of Péclet numbers used.

## Supplementary Material

pgad388_Supplementary_Data

## Data Availability

All code used to perform the simulations, code used for data analysis of the simulations, and data and scripts used to create the simulation figures in the main text and Supplementary material online are available in a public repository (56).
