# Supplementary material for: Goblet cell interactions reorient bundled mucus strands for efficient airway clearance
Source: PNAS Nexus. 2023 Nov 10;2(11):pgad388. doi: 10.1093/pnasnexus/pgad388 (PMC10661087; doi:10.1093/pnasnexus/pgad388)
Supplement: pgad388_Supplementary_Data [file pgad388_supplementary_data.zip › PNASNEXUS-PNASNEXUS-2023-01083-T-s01.docx]

**Supporting Information for**

Goblet-Cell Interactions Reorient Bundled Mucus Strands for Efficient Airway Clearance

Meike F. Bos, Anna Ermund, Gunnar C. Hansson, Joost de Graaf

Gunnar C. Hansson

Email: gunnar.hansson@medkem.gu.se

Joost de Graaf

Email: j.degraaf@uu.nl

**This PDF file includes:**

Supporting text

Figures S1 to S3

Table S1

Legends for Movies S1 and S2

**Other supporting materials for this manuscript include the following:**

Movies S1 and S2

Supporting Information Text

**Setup of the Simulations** In our simulations we used modified Péclet numbers to express all relevant forces in terms of the force experienced by the bead, due to the background flow, as described in the main text and Materials and Methods section. The magnitude of the force on a bead due to the background flow is given by:

$$F_{\text{flow}}=\left( 3\pi\eta\sigma_{b} \right)\sigma_{b}\dot{\gamma}=\xi v_{\text{flow}},$$

*(1)*

where the first group represents the friction. The combination $\sigma\dot{\gamma}$ represents the velocity difference between beads, induced by the shear rate $\dot{\gamma}$, which we estimate as $v_{\text{flow}}/\sigma_{b}$. The magnitude of the internal/external interaction forces is approximated by the spring constant/interaction strength times the length scale of the interaction force. In general, particles emerged in a fluid experience a thermal force due to collisions with the fluid. The magnitude this force is given by $\sqrt{2\xi k_{B}T}$. In Table S1 the length scales for all interaction forces in our model are given, as well as the range of Péclet numbers used. In our system, the thermal force is almost two orders of magnitude smaller than the background flow, and hence we neglect it.

For our simulations, we used a Euler forward scheme to integrate the equation of motions for all the beads as given by Eq. *(1)* in the main text. We used the modified Péclet numbers, introduced in the previous section to set the strength of all interactions. Since we use $\sigma_{b}$ as unit of length and $v_{\text{flow}}$ as unit of speed in our simulations, the unit of time is defined as $\Delta t=\sigma_{b}/v_{\text{flow}}$.

In each simulation we simulated a single strand. We used a square simulation box of size $200\sigma_{b}\times200\sigma_{b}$ with periodic boundary conditions. We rotated the direction of the fluid flow over an irregular angle $\alpha=\pi/\left( 5\sqrt{2} \right)$ with respect to the simulation box to reduce periodic effects. The random distribution of goblet cells on the surface was generated in a separate simulation, as we will discuss shortly. This generated distribution was used as input for our strand simulation.

As the exact trajectory of the strand is determined by all interactions it experiences over time, the measured values will be a function of the initial condition of the strand and the goblet cell configuration used. To measure the average behavior, we performed 50 simulations for each considered state point. For this, we used five different goblet cell configurations, and 10 different starting positions of the strand per goblet cell configuration.

We performed a 2D Monte Carlo (MC) simulation to generate the goblet cells configurations, given a specific area fraction thereof. We placed $N_{g}$ goblet cells (determined by the area fraction) randomly in a simulation box and let them interact through a repulsive Hookean-like pair potential

$$U_{\text{MC}}=\sum_{i=1}^{N_{g}} \sum_{j>i}^{N_{g}} \left\{ \begin{aligned} \varepsilon+10\varepsilon\frac{\sigma_{g}-r_{ij}}{\sigma_{g}},\quad\text{if}\quad r_{ij}<\sigma_{g}, \\ 0\quad\text{if}\quad r_{ij}\geq\sigma_{g} \end{aligned} \right.$$

*(2)*

where $r_{ij}$ is the distance between the center of disk *i* and *j*. The interaction strength is given by $\varepsilon$, which is expressed in unit of $k_{B}T$. The value of $\varepsilon$ was increased by $1k_{B}T$ after 100 mc-steps, if the energy was below $2N_{g}$, until zero overlaps where found. The final configuration was saved to a file, which was used as input file for the mucus strand simulation. It should be noted that the exact nature of the repulsion is not relevant to the final configuration, the above choice is merely a means, by which non-overlapping configurations could be achieved in a straightforward way.

**Definitions of Measured Quantities in the Simulations.** We measured the distance traveled, velocity, and orientation as function of system parameters. The distance traveled is directly given by the center of mass in the strand

$$R_{\text{CoM}}\left( t \right)=\frac{1}{N_{b}}\sum_{i=1}^{N_{b}} r_{i}\left( t \right).$$

*(3)*

The velocity of the bundled strand was quantified as the velocity of the center of mass of the strand. We use the velocity of the strand to measure whether a strand is trapped. In our simulations, there is no thermal noise, fluctuations in background flow, or interactions with other strands; hence, once a strand is trapped, it will remain trapped. We define the strand to be trapped when the velocity of the CoM is zero (the speed is < ${10}^{-6}v_{\text{flow}}$ numerically). When this criterion is reached, we terminate the simulation and log the trapped nature of the strand.

To quantify the shape of the strand, we measured the normalized cross sections of the strand $l_{\perp}$ and $l_{\parallel},$ which measure the length of the stand along the direction perpendicular or parallel to the flow, respectively. These observables are defined as:

$$l_{\parallel}=\frac{\text{max}\{y_{i}\}-\text{min}\{y_{i}\}}{\left( N_{b}-1 \right)\sigma_{b}};$$

$$l_{\perp}=\frac{\text{max}\{x_{i}\}-\text{min}\{x_{i}\}}{\left( N_{b}-1 \right)\sigma_{b}}.$$

*(4)*

Here, $\text{min}/\text{max}\{\alpha_{i}\}$ denotes the min or maximum value of the *x* or *y* coordinate taken from all $N_{b}$ beads comprising the strand. The distance between the maximum and minimum value of *x* or *y* is normalized by the length found, had the strand been completely straight in this direction. In Fig. 3A of the main text, we showed a series of snapshots of the simulated mucus bundles as function of interaction strength, where the shape fluctuations grow with increasing $\text{P}\text{e}_{\text{g}}$. The amplitude of the shape fluctuations can be quantified by $l_{\parallel}$ and the variations thereof as shown in Fig. S2.

We measured the impulse due to a single goblet cell interacting with single bead of a chain for seven configurations. The impulse is defined as the time integral over the force$F_{g}^{y}\left( t \right)$

$$\text{Impulse}=\int_{0}^{T} F_{g}^{y}\left( t \right)dt,$$

*(5)*

where T is the measurement period, which we define as the time when the interaction force is nonzero. Here, we defined $t=0$ as the moment when the bead experiences a nonzero force due to the goblet cell for the first time, *i.e.*, when the interaction kicks in.


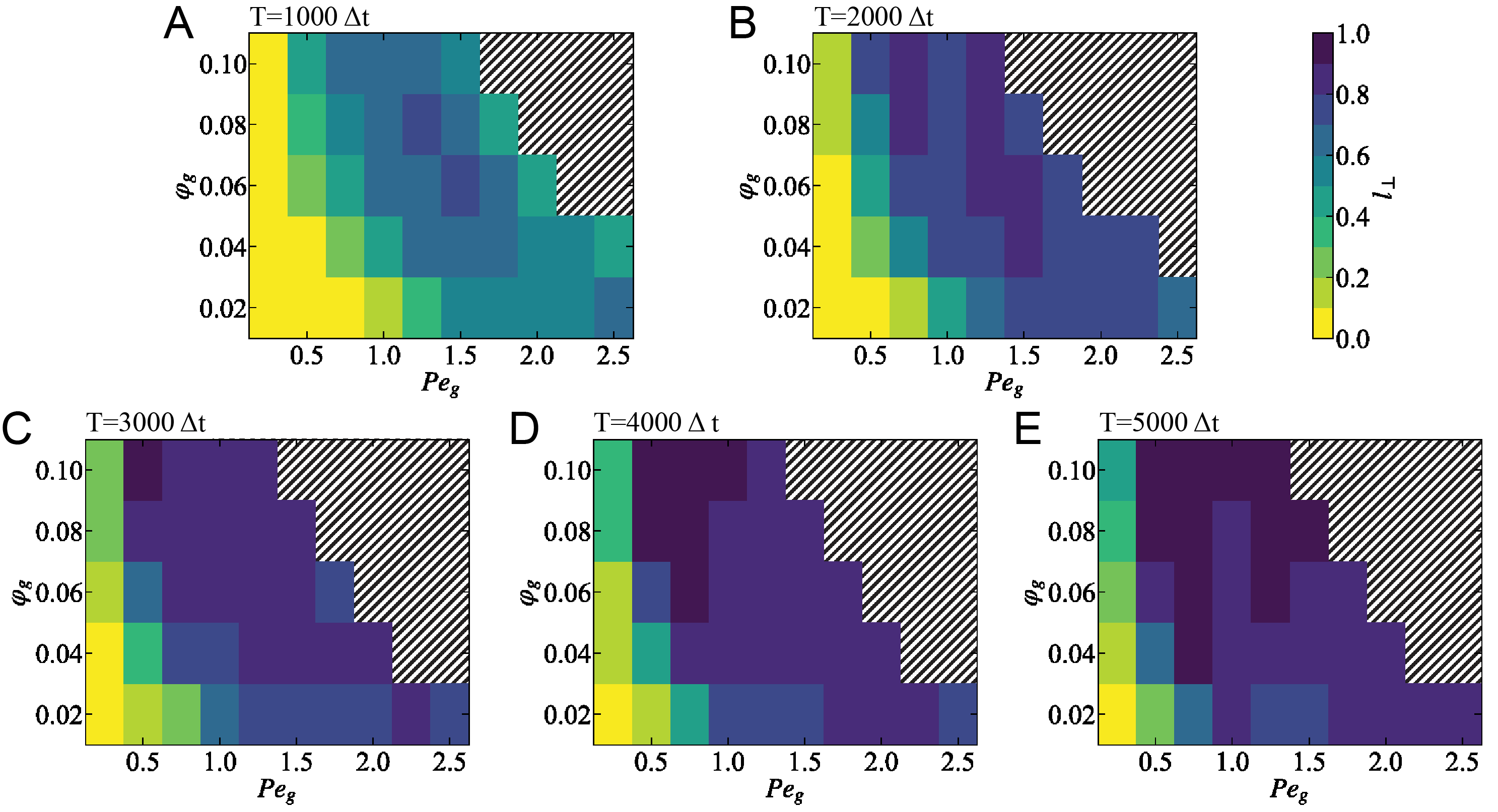


Fig. S1. State diagram of $\boldsymbol{l}_{\boldsymbol{\perp}}$ for range of times. The graphs show the average value over 50 simulations of $\boldsymbol{l}_{\boldsymbol{\perp}}$(top-left label for each panel) for (A) $T=1000\sigma_{b}/v_{\text{flow}}$, (B) $T=2000\sigma_{b}/v_{\text{flow}}$, (C) $T=3000\sigma_{b}/v_{\text{flow}}$, (D) $T=4000\sigma_{b}/v_{\text{flow}}$, (E) $T=5000\sigma_{b}/v_{\text{flow}}$, where the value is averaged over 50 different runs. The value of $l_{\perp}$ is indicated by the color as given in the top-right legend.


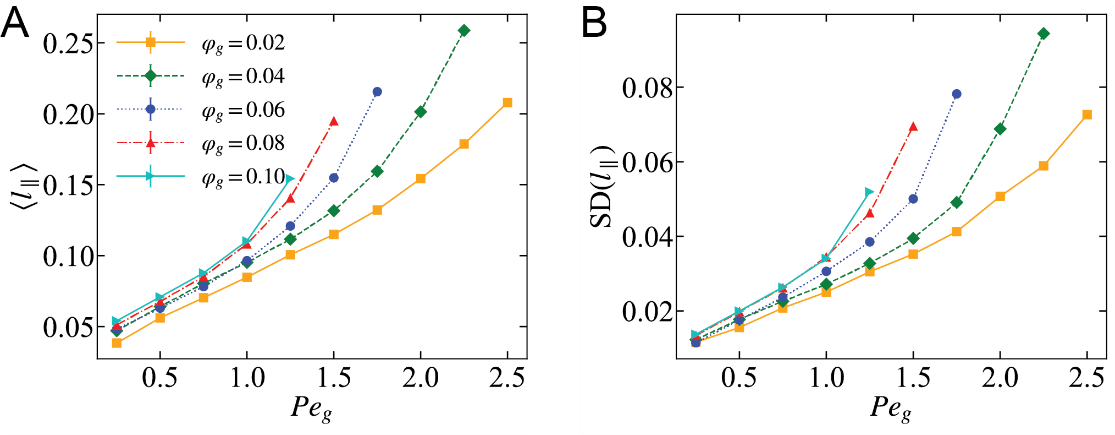


Fig. S2. The shape and fluctuations thereof for a perpendicular moving strand. (A) Average steady-state parallel cross section $\boldsymbol{l}_{\boldsymbol{\parallel}}$ for strands starting with a perpendicular orientation. The values are shown for different goblet-cell area fractions $\boldsymbol{\varphi}_{\boldsymbol{g}}$ (see the legend), as function of goblet-cell interaction strength $\text{P}\text{e}_{\text{g}}$. The average per point is taken over 50 simulations. The error bars (smaller than the data points) represent the SEM. (B) Standard deviation (SD) for the same data as shown in (A). The error bars (smaller than the data points) represent the SEM.


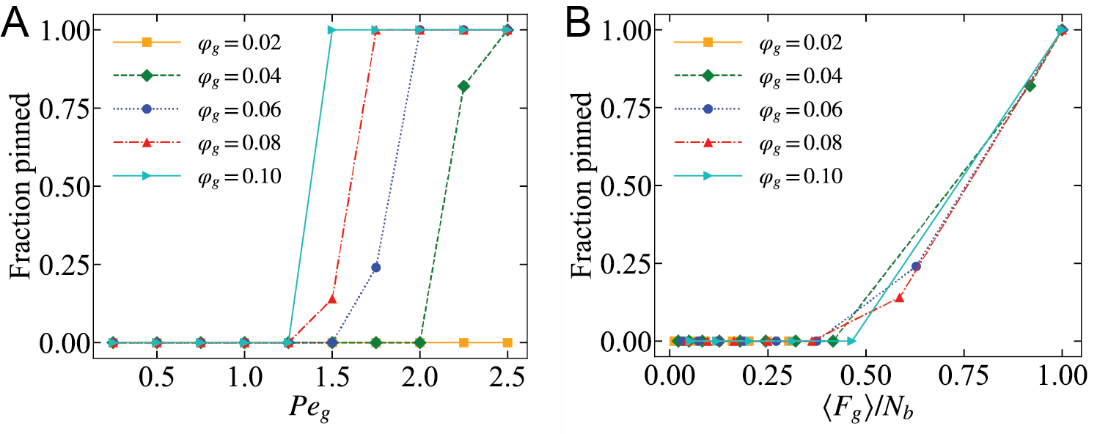


Fig. S3. Pinning transition in bundled mucus strands. (A) Fraction of 50 simulations that were pinned after $\boldsymbol{T=2000}\boldsymbol{\sigma}_{\boldsymbol{b}}\boldsymbol{/}\boldsymbol{v}_{\text{flow}}$ for different goblet-cell area fractions $\boldsymbol{\varphi}_{\boldsymbol{g}}$ as function of the goblet cell interaction strength $\text{P}\text{e}_{\text{g}}$. (B) Same data as function of mean total goblet cell force on the strand $\left\langle\boldsymbol{F}_{\boldsymbol{g}} \right\rangle$ normalized by the number of beads $\boldsymbol{N}_{\boldsymbol{b}}$.


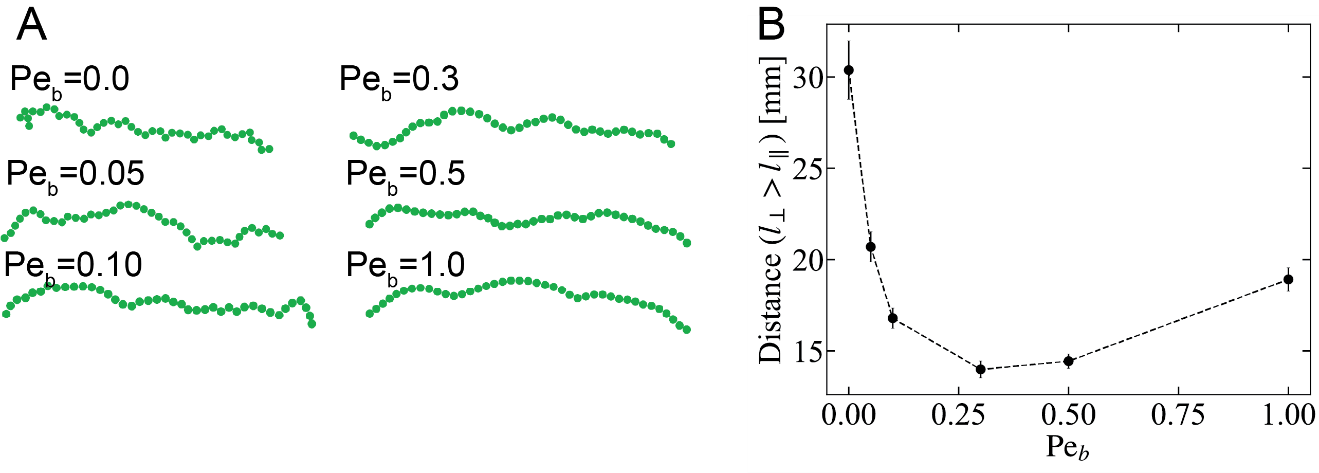

Fig. S4. Strand dynamics as function of bending stiffness. We used $\boldsymbol{\varphi}\boldsymbol{=0.04}$ and $\text{P}\text{e}_{\text{g}}\boldsymbol{=1.5}$. (A) Characteristic shapes found in steady state for different values of $\text{P}\text{e}_{\text{b}}$ as indicated above each snapshot. (B) Average turning distance as function of $\text{P}\text{e}_{\text{b}}$, our selection corresponds to the approximate minimum in this distance.

| Force | Length scale | Magnitude | Pe |
| --- | --- | --- | --- |
| $F_{\text{fluid}}$ | $\sigma_{b}$ | $\xi v_{\text{flow}}$ | 1 |
| $F_{\text{bending}}$ | $\sigma_{b}$ | $k_{\text{bend}}/\sigma_{b}$ | 0.3 |
| $F_{\text{spring}}$ | $\Delta r_{\text{max}}=0.8\sigma_{b}$ | $k_{\text{spring}}\Delta r_{\text{max}}$ | 5 |
| $F_{\text{overlap}}$ | $\sigma_{b}$ | $E_{r}\sigma_{b}$ | 1 |
| $F_{\text{goblet}}$ | $r_{\text{bond}}=0.75\sigma_{b}$ | $k_{\text{g}}r_{\text{bond}}$ | 0.25-2.5 |
| $F_{\text{thermal}}$ | $\sigma$ | $\sqrt{2\xi k_{B}T}$ | $6\cdot{10}^{-2}$ |

Table S1. Modified Péclet number for all forces in the system. The modified Péclet numbers follow from the estimates for the length scale and magnitude reported here.

Movie S1 (separate file). Alcian blue stained bundled mucus strand transport on an explanted weaned pig trachea, duration 5 min, 16x normal speed. Extracted images in Fig. 1A.

Movie S2 (separate file). Movie of the turning event for $\boldsymbol{\varphi}_{\boldsymbol{g}}\boldsymbol{=0.04}$ and $\boldsymbol{P}\boldsymbol{e}_{\boldsymbol{g}}\boldsymbol{=2}$ in the center-of-mass frame of the mucus strand as shown in Fig. 1B. The flow points upward. The green beads represent the mucus strand. The grey circles indicate the goblet cells. There are 20 frames per second, each frame is taken $\boldsymbol{\sigma}_{\boldsymbol{b}}\boldsymbol{/}\boldsymbol{v}_{\text{flow}}$ apart. The total simulation time is $\boldsymbol{1000}\boldsymbol{\sigma}_{\boldsymbol{b}}\boldsymbol{/}\boldsymbol{v}_{\text{flow}}$
